# The impact of early morphine administration on septic patients with pre-existing chronic heart failure

**DOI:** 10.1016/j.clinsp.2025.100655

**Published:** 2025-04-24

**Authors:** Zhi-Ye Zou, Shui-Qing Gui

**Affiliations:** Department of Critical Care Medicine, Shenzhen Second People's Hospital & First Affiliated Hospital of Shenzhen University, Shenzhen, China

**Keywords:** Sepsis, Chronic heart failure, MIMICIV database, Effect, Morphine

## Abstract

**Background:**

The use, timing, and dosage of morphine in septic patients with a history of Chronic Heart Failure (CHF) are poorly studied, and it is still unclear if morphine administration affects sepsis survival. This study aims to elucidate the relationship between early morphine administration and 30-day mortality among septic patients with a history of CHF.

**Methods:**

Utilizing data from the Medical Information Mart for Intensive Care IV (MIMIC-IV) database, this retrospective cohort study focuses on septic patients with CHF. The authors investigated the effects of early morphine administration (within 24 h of ICU admission) on 30-day mortality, employing Cox proportional hazards modeling and Propensity Score Matching (PSM) for analysis.

**Results:**

The authors analyzed 7424 adult septic patients with CHF, among whom 6495 received early morphine and 929 were managed without morphine. The early morphine group exhibited a significantly lower 30-day mortality rate compared to the nonmorphine group (post-matched 16.4 % vs. 25.7 %, Absolute Risk Reduction (ARR) 9.3 % (95 % Confidence Interval [95 % CI 5.1–13.6], p < 0.001). Among septic patients with CHF, early morphine administration was associated with a reduction in 30-day death in all adjusted variables (HR = 0.539‒0.735, p < 0.001). After PSM, only <15 mg of morphine was correlated to a lower 30-day mortality rate (HR = 0.233, 95 % CI 0.086–0.632, p < 0.001).

**Conclusion:**

This investigation reveals a significant association between the early administration of morphine and reduced risk-adjusted 30-day mortality in septic patients with CHF.

## Highlights

• Early Morphine in Septic CHF Patients Reduces 30-Day Mortality (HR = 0.539).
• Morphine Dose < 15 mg Lowers Mortality in Septic CHF Patients (HR = 0.233).
• Analysis of 7424 Septic CHF Patients from MIMIC-IV Database.

## Introduction

Chronic Heart Failure (CHF) emerges as a significant comorbidity in hospitalized septic patients, ranking second only to cancer in prevalence.^1^^,^^2^ This association is particularly concerning given that sepsis is implicated in over half of the noncardiovascular fatalities among individuals with CHF.^3^^,^^4^ Managing septic patients with CHF may be difficult since common therapies for the condition, including diuretic therapy, may not be appropriate in sepsis and vice versa.^5^ Despite the critical nature of this intersection between sepsis and CHF, the medical community lacks specific guidelines, leaving clinicians to rely on empirical strategies for patient care.^6^

Morphine is traditionally employed in the management of both acute and chronic heart failure due to its analgesic and vasodilatory properties,^7^ but it is also controversial.^8^^,^^9^ Several studies have even pointed out that morphine can induce sepsis,^10^ and even increase mortality.^11^ There is no literature report on the use of morphine to treat patients with new-onset sepsis who have a history of CHF.

This study aims to bridge this knowledge gap by examining the impact of morphine administration on 30-day mortality in patients with concurrent CHF and sepsis. Specifically, the authors scrutinize the role of early morphine intervention within the first 24 h following ICU admission in influencing the survival outcomes of septic patients with pre-existing CHF. Through this retrospective cohort analysis, the authors seek to elucidate the relationship between early morphine administration and 30-day mortality, thereby contributing valuable insights to the ongoing discourse on the management of sepsis in patients with CHF.

## Methods

### Data source and study design

The authors used data from Medical Information Mart for Intensive Care IV (MIMIC-IV) version 2.0 (https://physionet.org/content/mimiciv/2.0/), which improved upon MIMIC Ⅲ by adding partial table reconstruction and data updates. The authors conducted a retrospective cohort analysis utilizing this resource. 450,000 hospitalization records that were admitted to the Beth Israel Deaconess Medical Centre (BIDMC) between 2008 and 2019, as well as the clinical data of over 190,000 patients, were gathered. The database is accessible to those who successfully pass the Collaborative Institutional Training Initiative test (author ZYZ's certification number is 59,729,494). De-identification was carried out to protect patient privacy. The Shenzhen Second People's Hospital Research Ethics Committee gave its approval to this study (2024-145-01PJ). The report complies with the recommendations of STROBE (Strengthening the Reporting of Observational Studies in Epidemiology).

### Participants

The inclusion criteria were patients who met sepsis 3.0 diagnostic criteria and had chronic heart failure before ICU admission. Sepsis was defined as having a Sequential Organ Failure Assessment (SOFA) score of ≥ 2 and a proven or suspected infection.^12^ Patients with exertional or paroxysmal nocturnal dyspnea who have responded symptomatically (or on physical examination) to digitalis, diuretics, or afterload-reducing medications are classified as having Chronic Heart Failure (CHF). Patients using one of those drugs but not responding to treatment or showing any improvement in their physical symptoms are not included. The extraction code for CHF is substr (icd9_code, 1, 3) = '428′ or substr (icd9_code, 1, 5) in ('39,891′, '40,201′, '40,211′, '40,291′, '40,401′, '40,403′, '40,411′, '40,413′, '40,491′, '40,493′) or substr (icd9_code, 1, 4) between '4254′ and '4259′ or substr (icd10_code, 1, 3) in ('I43′, 'I50′) or substr (icd10_code, 1, 4) in ('I099′, 'I110′, 'I130′, 'I132′, 'I255′, 'I420′, 'I425′, 'I426′, 'I427′, 'I428′, 'I429′, 'P290′).

Ages under 18 and over 90, stay in the Intensive Care Unit (ICU) for <24 h, death in the ICU within 24 h, and a secondary tumor diagnosis at the time of admission were the exclusion criteria. The authors only used the first ICU admission data from the initial hospital stay for patients who were hospitalized in the ICU more than once.

### Research procedures and definitions

Structured Query Language with Navicat Premium (version 12.0.28) was used to retrieve the data from MIMIC-IV. Early morphine agents referred to the use of morphine drugs within 24 h after ICU admission. The routes of morphine administration included oral, intravenous, and aerosol administration. Later morphine agent use referred to the use of morphine after admission to the ICU for 24 h.

The International Classification of Diseases, Ninth Revision (ICD-9), or ICD-10 codes (icd10_code or icd9_code) supplied in the MIMIC-IV database were used to identify the infection location. In-hospital management (including renal replacement therapy and mechanical ventilation) and medications (furosemidum, dexmedetomidine, fentanyl, midazolam, and propofol) referred to the use of related treatment methods within 24 h of admission to the ICU. The maximum sequential organ failure assessment (SOFA) score, maximum simplified acute physiology score II (SAPII) score, minimum Glasgow Coma Scale (GCS) score, vital signs, and laboratory outcomes referred to the results obtained within the first 24 h of ICU admission. The Minimum Left Ventricular Ejection Fraction (LVEF) is the lowest value during the ICU stay.

### Exposure and outcomes

Patients were divided into two groups: those who received no morphine (0–24 h after ICU admission) and those who received early morphine (0–24 h after ICU admission combined). The 30-day all-cause mortality was the primary outcome. The secondary outcomes were the length of hospital stay, ICU stay, and 90-day all-cause mortality.

### Statistical analysis

For each set of data, the appropriate presentation is made as the mean ± standard deviation (mean ± SD), median and Interquartile Range (median, IQR), or percentage (n %). The χ^2^ test and Student's *t*-test were used, where applicable, to analyze the differences between the groups. While a small number of variables had <2 % missing data, the majority of variables had no missing values. Since missing values were assumed to be absent at random, multiple imputation was applied (Supplementary Table S1).

Confounding factor balancing was achieved through the use of propensity score matching (PSM). All of the factors in Table 1 were included in the PS because they were selected for their clinical significance and prior research. A multivariable logistic regression model was built by us. A caliper width of 0.02 was used in conjunction with a one-to-one closest neighbor matching technique. Standardized Mean Differences (SMDs) and p-values were utilized after PSM to assess how evenly the groups' baseline attributes were distributed. An imbalance between groups was taken into consideration when a variable's SMD was >0.1 (Supplementary Fig. S1). Ultimately, 684 patients in each group met the criteria for matching, and their information was taken out for additional study. Kaplan-Meier and log-rank tests were used for survival analysis both before and after PSM. Furthermore, the authors computed the Absolute Risk Reduction (ARR) for 30-day mortality based on whether or not early morphine was used.

**Table 1:** Baseline demographics and clinical characteristics of the patients with no morphine group and early morphine group.

	**Propensity score matching**							
	**Before**				**After**			
	**All patients** **(n = 7424)**	**No morphine** **(n = 6495)**	**Early morphine** **(n = 929)**	**p-value**	**No morphine** **(n = 684)**	**Early morphine** **(n = 684)**	**p-value**	**SMD**
**Baseline characteristics**								
Age (year), median (IQR)	72.4 (62.7, 80.7)	72.3 (62.7, 80.8)	73.4 (63.3, 80.3)	0.75	73.4 (63.9, 81.1)	73.6 (63.8, 80.5)	0.81	<0.001
Male, n (%)	4207 (56.7)	3674 (56.6)	533 (57.4)	0.64	369 (53.9)	375 (54.8)	0.74	0.018
White, n (%)	4974 (67.0)	4300 (66.2)	674 (72.6)	<0.001	484 (70.8)	485 (70.9)	0.95	0.003
Insurance, Medicare, n (%)	4440 (59.8)	3880 (59.7)	560 (60.3)	0.75	429 (62.7)	419 (61.3)	0.58	0.030
Weight (kg), median (IQR)	81.9 (67.9, 98.4)	81.8 (67.9, 98.5)	82.1 (68.3, 98.0)	0.80	83.0 (67.3, 99.5)	81.0 (66.9, 97.4)	0.40	0.032
Admission (emergency), n (%)	3503 (47.2)	3203 (49.3)	300 (32.3)	<0.001	271 (39.6)	270 (39.5)	0.96	0.003
**History of disease, n (%)**								
Hypertension	5957 (80.2)	5241 (80.7)	716 (77.1)	0.010	537 (78.5)	528 (77.2)	0.56	0.032
Myocardial infarction	2399 (32.3)	2093 (32.2)	306 (32.9)	0.66	213 (31.1)	221 (32.3)	0.64	0.025
Diabetes	3506 (47.2)	3119 (48.0)	387 (41.7)	<0.001	274 (40.1)	294 (43.0)	0.27	0.059
Renal disease	3334 (44.9)	3031 (46.7)	303 (32.6)	<0.001	258 (37.7)	253 (37.0)	0.78	0.015
Chronic pulmonary disease	3030 (40.8)	2632 (40.5)	398 (42.8)	0.18	315 (46.1)	295 (43.1)	0.28	0.059
Charlson comorbidity index, mean (SD)	7.4 (2.3)	7.5 (2.3)	6.9 (2.2)	<0.001	7.1 (2.2)	7.1 (2.3)	0.65	0.024
**Infection sites, n (%)**								
Respiratory infection	3229 (43.5)	3040 (46.8)	189 (20.3)	<0.001	197 (28.8)	180 (26.3)	0.30	0.056
Urinary tract infection	1520 (20.5)	1371 (21.1)	149 (16.0)	<0.001	127 (18.6)	128 (18.7)	0.94	0.004
Bloodstream infection	619 (8.3)	573 (8.8)	46 (5.0)	<0.001	41 (6.0)	43 (6.3)	0.82	0.012
Abdominal infection	396 (5.3)	353 (5.4)	43 (4.6)	0.31	38 (5.6)	40 (5.8)	0.82	0.013
**Vital signs at 1^st^ day, median (IQR)**								
Mean MAP (mmHg)	73.9 (68.2, 80.3)	74.0 (68.3, 80.6)	73.3 (67.9, 78.5)	0.003	73.5 (68.1, 79.7)	73.6 (68.1, 79.0)	0.89	0.024
Maximum heart rate (bpm)	102.0 (89.0, 118.0)	103.0 (89.0, 119.0)	100.0 (89.0, 114.0)	0.009	103.0 (90.0, 119.0)	101.5 (90.0, 117.0)	0.59	0.013
Maximum respiratory rate (bpm)	28.0 (25.0, 33.0)	28.0 (25.0, 33.0)	28.0 (24.0, 32.0)	<0.001	28.0 (24.0, 33.0)	28.0 (24.0, 32.0)	0.79	0.002
**Laboratory outcomes at 1^st^ day, median (IQR)**								
Maximum white blood cell (10^9/L)	13.6 (9.9, 18.7)	13.4 (9.7, 18.4)	15.2 (11.0, 20.0)	<0.001	14.1 (10.1, 19.4)	14.5 (10.7, 19.5)	0.36	0.026
Maximum platelets(10^9/L)	211.0 (155.0, 283.0)	214.0 (156.0, 287.0)	197.0 (151.0, 257.0)	<0.001	200.0 (144.0, 275.0)	206.0 (154.0, 269.5)	0.25	0.051
Septic shock, n (%)	2617 (35.3)	2382 (36.7)	235 (25.3)	<0.001	187 (27.3)	185 (27.0)	0.90	0.007
**Scoring system at 1^st^ day, median (IQR)**								
Maximum SOFA score	7.0 (4.0, 9.0)	7.0 (4.0, 10.0)	6.0 (4.0, 9.0)	0.013	7.00 (4.00, 9.00)	6.00 (4.00, 9.00)	0.14	0.090
Maximum SAPII score	40.0 (33.0, 49.0)	40.0 (33.0, 49.0)	39.0 (32.0, 48.0)	<0.001	40.0 (33.0, 49.0)	39.0 (32.0, 48.0)	0.17	0.060
Minimum GCS score	13.0 (9.0, 14.0)	13.0 (9.0, 14.0)	14.0 (10.0, 15.0)	0.004	13.0 (9.0, 15.0)	14.0 (10.0, 15.0)	0.31	0.049
**In-hospital management at 1^st^ day, n (%)**								
Mechanical ventilation	3406 (45.9)	2907 (44.8)	499 (53.7)	<0.001	338 (49.4)	307 (44.9)	0.093	0.091
Renal replacement therapy	768 (10.3)	727 (11.2)	41 (4.4)	<0.001	39 (5.7)	36 (5.3)	0.72	0.019
**In-hospital medication at 1^st^ day, n (%)**								
Furosemidum	3032 (40.8)	2584 (39.8)	448 (48.2)	<0.001	320 (46.8)	319 (46.6)	0.96	0.003
Dexmedetomidine	407 (5.5)	303 (4.7)	104 (11.2)	<0.001	45 (6.6)	53 (7.7)	0.40	0.045
Fentanyl	2714 (36.6)	2619 (40.3)	95 (10.2)	<0.001	117 (17.1)	94 (13.7)	0.085	0.093
Midazolam	1492 (20.1)	1416 (21.8)	76 (8.2)	<0.001	78 (11.4)	69 (10.1)	0.43	0.042
Propofol	2843 (38.3)	2250 (34.6)	593 (63.8)	<0.001	386 (56.4)	358 (52.3)	0.13	0.082
Later morphine agent, n (%)	1209 (16.3)	830 (12.8)	379 (40.8)	<0.001	249 (36.4)	228 (33.3)	0.23	0.064
Minimum LVEF	45.0 (31.0, 55.0)	45.0 (30.0, 55.0)	45.0 (35.0, 55.0)	0.44	45.0 (30.0, 55.0)	45.0 (35.0, 55.0)	0.10	0.030

SMD, Standardized Mean Difference; IQR, Interquartile Range; SD, Standard Deviation; MAP, Mean Blood Pressure; bpm, beat per minute or breaths per minute; SOFA, Sequential Organ Failure Assessment; SAPII, Simplified Acute Physiology score-II; GCS, Glasgow Coma Scale; LVEF, Left Ventricular Ejection Fraction.

Covariates that could have an impact on the results were taken into account using an expanded Cox model. To find out if there are differences in morphine administration and 30-day mortality between subgroups categorized by maximal SOFA, septic shock, mechanical ventilation, propofol, fentanyl, midazolam, and later morphine agent, subgroup and interaction analyses were performed. A Cox model that had been adjusted for every variable in the patient baseline data was also employed for subgroup analysis.

To investigate the connection between early morphine treatment, later morphine treatment, minimum LVEF, and 30-day mortality further, univariate analyses were carried out, and variables with p < 0.1 were included in multivariate analysis.

Statistical significance was defined as two-tailed p-values <0.05. R 4.0.1 for Windows and Stata 15.1 (StataCorp, College Station, TX, United States) were used for all statistical analyses.

## Results

### Baseline characteristics

**Fig. 1:**
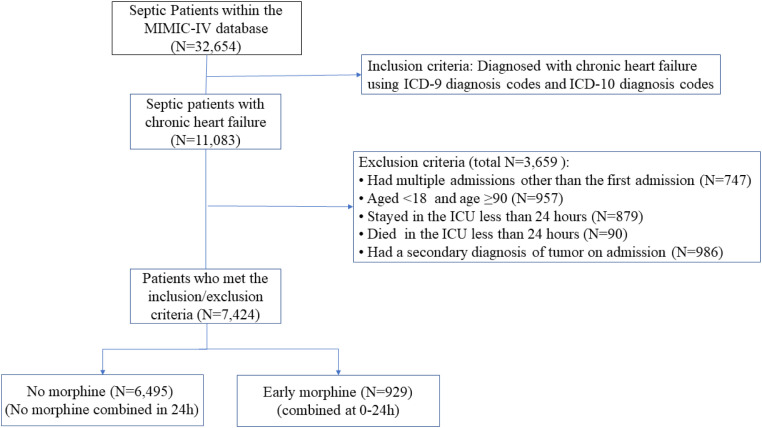
Flowchart of patient selection.

This study evaluated data from 11,083 septic patients with a history of CHF, ultimately including 7424 patients in the analysis (Fig. 1). Of these, 929 received morphine within the first 24 h, while 6495 did not. Supplementary Table 1 outlines the presence of missing values across variables.

In total, 7424 septic patients with a history of chronic heart failure were included in this study (Table 1). The median (IQR) age was 72.4 (62.7, 80.7) years, 56.7 % were male, 67.0 % were white, and 16.3 % of patients used or continued to use morphine after 24 h. The no morphine group had significantly higher emergency admission, hypertension, diabetes, renal disease, Charlson comorbidity disease, sofa, fentanyl, and midazolam on the first day than that in the early morphine group. Compared to the no morphine group, the early morphine group was more likely to have had dexmedetomidine, propofol, and continue to use morphine after 24 h (40.8 vs. 12.8 %, p < 0.001). The 30-day mortality rate in the early morphine group was not significantly different than that in the nonmorphine group (pre-matched 45.0 vs. 45.0, post-matched 45.0 vs. 45.0, p > 0.05).

### Outcomes

In PSM, 684 pairs of patients were matched by a 1:1 matching algorithm (Table 1 and Supplementary Fig. 1). There was no significant difference between the two groups after PSM, and all SMDs were < 0.1.

The pre-matched 30-day mortality rate was significantly lower in patients with early morphine use than in those without morphine use (13.1 vs. 21.5 %, p < 0.001). After PSM, similar to the results in the pre-matched model, morphine was associated with reduced 30-day mortality (16.4 % vs. 25.7 %, absolute risk reduction 9.3 [95 % CI 5.1–13.6], p < 0.001). The 90-day mortality rate in the early morphine group was lower than that in the nonmorphine group (pre-matched 18.0 vs. 30.7 %, post-matched 22.5 % vs. 32.7 %, p < 0.01) (Table 2).

**Table 2:** Primary and secondary outcome results before and after PSM.

	**Propensity score matching**								
		**Before**				**After**			
**Variables**	**All patients** **(n = 7424)**	**No morphine** **(n = 6495)**	**Early morphine** **(n = 929)**	**ARR (95 %CI)**	**p-value**	**No morphine** **(n = 684)**	**Early morphine** **(n = 684)**	**ARR (95 %CI)**	**p-value**
Primary outcome									
30-day mortality, n (%)	1518 (20.4)	1396 (21.5)	122 (13.1)	8.4 (6.0, 10.8)	<0.001	176 (25.7)	112 (16.4)	9.3 (5.1, 13.6)	<0.001
Secondary outcomes									
In-hospital mortality, n (%)	1226 (16.5)	1124 (17.3)	102 (11.0)	6.3 (4.1, 8.5)	<0.001	147 (21.5)	95 (13.9)	7.6 (3.6, 11.6)	<0.001
90-day mortality, n (%)	2163 (29.1)	1996 (30.7)	167 (18.0)	12.7 (10,0, 15.5)	<0.001	224 (32.7)	154 (22.5)	10.2 (5.5, 14.9)	<0.001
Length of ICU stay (days), median (IQR)	3.9 (2.2, 7.3)	4.0 (2.2, 7.6)	3.2 (2.0, 5.5)	NA	<0.001	4.2 (2.3, 7.8)	3.2 (2.1, 5.6)	NA	<0.001
Length of hospital stay(days), median (IQR)	10.3 (6.5, 17.0)	10.6 (6.6, 17.6)	9.2 (6.1, 13.8)	NA	<0.001	10.2 (6.4, 16.2)	9.0 (6.0, 13.8)	NA	<0.001

PSM, Propensity Score Matching; ARR, Absolute Risk Reduction; ICU, Intensive Care Unit; IQR, Interquartile Range; NA, Not Application.

**Fig. 2:**
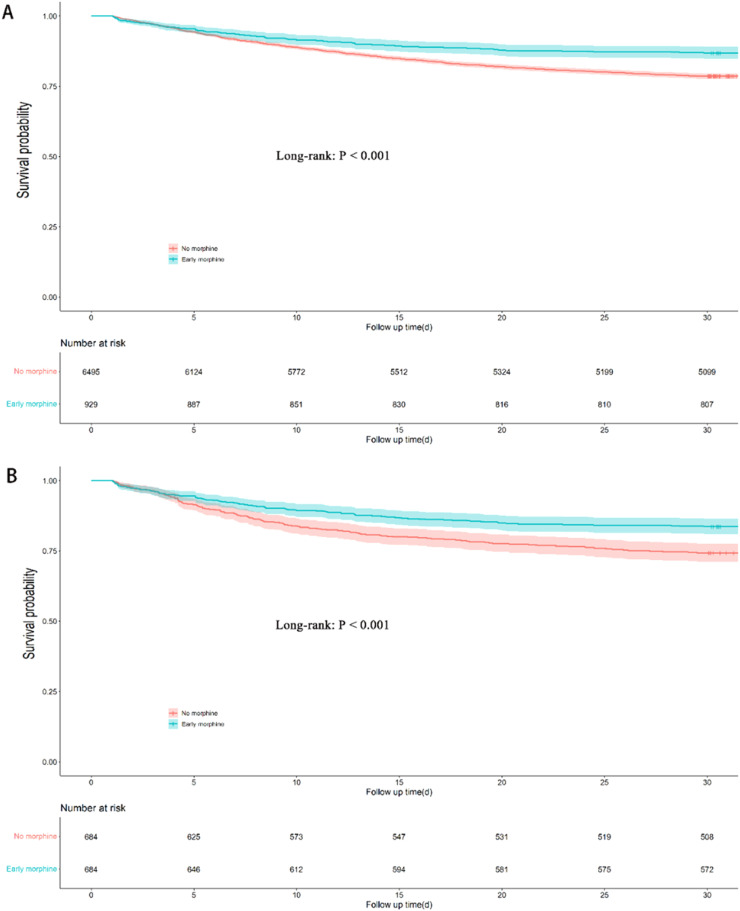
Kaplan-Meier survival curve of two groups before (A) and after (B) PSM.

In the extended multivariable Cox proportional hazards models, HR of early morphine use was consistently significant in five models after adjustment for covariates (HR range 0.539–0.735, all p < 0.001) (Table 3). The Kaplan-Meier curves showed a significant difference between early morphine use and non-morphine use before and after PSM (p < 0.001) (Fig. 2A and B).

**Table 3:** Efficacy of early morphine therapy in 30-day mortality and dose used.

**Variables**	**Hazard ratio**	**95 % CI**	**p-value**
Model 1	0.591	0.491‒0.711	<0.001
Model 2	0.600	0.498‒0.723	<0.001
Model 3	0.735	0.608‒0.887	<0.001
Model 4	0.713	0.590‒0.862	<0.001
Model 5	0.539	0.440‒0.660	<0.001
Early morphine use (mg^a^/24 h)			
No morphine use	1.000 (reference)		
**Pre-matched cohort**			
1–5	0.791	0.633‒0.989	0.040
6–10	0.248	0.154‒0.401	<0.001
11–15	0.138	0.051‒0.370	<0.001
16–20	0.433	0.192‒0.979	0.044
≥ 21	0.721	0.319‒1.630	0.431
P for trend	<0.001		
**Post-matched cohort**			
1–5	0.608	0.456‒0.810	0.001
6–10	0.331	0.200‒0.549	<0.001
11–15	0.233	0.086‒0.632	0.004
16–20	0.572	0.208‒1.573	0.279
≥ 21	1.611	0.677‒3.833	0.281
P for trend^b^	<0.001		

Adjusted covariates: Model 1 = early morphine use. Model 2 = Model 1 + Age, Male, White, Insurance, Weight, Admission (emergency). Model 3 = Model 2 + (history of disease including Hypertension, Myocardial infarction, Diabetes, Renal disease, Chronic pulmonary disease, Charlson comorbidity index) + (Infection sites including Respiratory infection, Urinary tract infection, Bloodstream infection, Abdominal infection). Model 4 = Model 3 + Septic shock + Vital signs, Laboratory outcomes, and Scoring system on 1^st^ day. Model 5 = Model 4 + Later morphine agent + In-hospital management and medication on 1^st^ day.

a mg, the sum of intravenous, oral, and nasogastric morphine doses in 24 h after ICU admission.

b P for interaction was < 0.001, indicating that there was a significant difference in the relationship between early morphine different doses on the first day and 30-day death.

Before PSM, the patients with a dose of <20 mg on the first day were associated with 30-day mortality. After PSM, the patients with a dose of <15 mg were associated with 30-day mortality (HR = 0.233, 95 % CI 0.086–0.632, p < 0.001), with a P for trend of <0.001 (Table 3).

The length of stay in ICU (3.2 vs. 4.0 days, p < 0.001) and hospital (9.2 vs. 10.6 days, p < 0.001) were significantly shorter in the early morphine group than in the no morphine group (Table 2).

Significant known and measured risk factors for 30-day mortality within the multivariable Cox proportional hazard model included age (HR = 1.026 [95 % CI 1.019–1.034]), Charlson comorbidity index (HR = 1.100 [95 % CI 1.054–1.149]), respiratory infection (HR = 1.242 [95 % CI 1.079–1.428]), maximum heart rate (HR = 1.003 [95 % CI 1.000–1.007]), maximum respiratory rate (HR = 1.021 [95 % CI 1.011–1.031]), maximum SOFA score (HR = 1.124 [95 % CI 1.088–1.162]), later morphine agent (HR = 2.875 [95 % CI 2.473–3.344]) (Table S2). Significant measured protective factors for 30-day mortality included weight (HR = 0.994 [95 % CI 0.0.991–0.997]), minimum GCS score (HR = 0.953 [95 % CI 0.934–0.973]), mechanical ventilation (HR = 0.639 [95 % CI 0.507–0.806]), early propofol (HR = 0.709 [95 % CI 0.587–0.856]), and early morphine use (HR = 0.543 [95 % CI 0.418–0.707]) (Table S2).

### Subgroup analysis

**Fig. 3:**
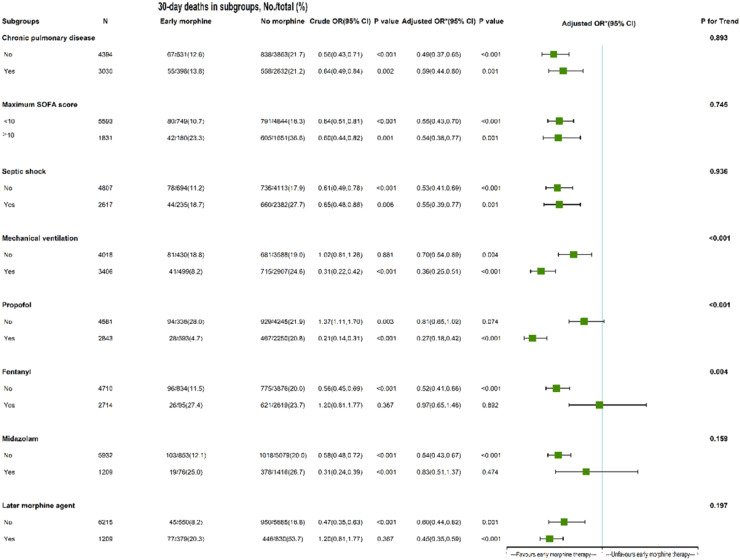
The role of early morphine therapy in subgroups of septic patients with CHF. *The authors adjusted age, gender, ethnicity, insurance, weight, source of admission, history of the disease, charlson comorbidity index, Infection sites, vital signs at 1^st^ day, laboratory outcomes at 1^st^ day, septic shock, the scoring system at 1^st^ day, maximum SOFA score, maximum SAPII score, minimum GCS score, in-hospital management at 1^st^ day, in-hospital medication at 1^st^ day, and later morphine agent. The last column's P for interaction value of 0.893 showed that there was no statistically significant variation in the association between early morphine treatment and 30-day mortality in patients with chronic lung disease compared to those who did not. In other words, the previously observed variations in HR of 0.49 and 0.59 were not statistically significant, and there was not a significant distinction in the association between early morphine administration and 30-day mortality between patients with and without chronic pulmonary illness. The interpretation of other measures, such as the maximal SOFA score, septic shock, and later morphine agent, is comparable to those of groups with chronic pulmonary illness. Conversely, P for interaction was <0.001, indicating that the association between early morphine treatment and 30-day mortality differed significantly between the groups receiving mechanical ventilation and those not.

In the early propofol cohort, early morphine therapy was substantially associated with lower 30-day mortality (adjusted HR = 0.27, 95 % CI 0.18–0.42), but not in the non-propofol category. Conversely, early morphine therapy was not related to decreased 30-day mortality in the early fentanyl and early midazolam usage group, but it was in the non-fentanyl and non-midazolam subgroup (adjusted HR = 0.52, 95 % CI 0.41–0.66; 0.54, 95 % CI 0.43‒0.67, respectively). The association between early morphine treatment and 30-day mortality remained significant in other subgroups (with or without chronic pulmonary disease, septic shock, mechanical ventilation, later morphine agent, and any sofa score). The mechanical ventilation subgroup had a P for trend of <0.001, and the HR differences of 0.70 and 0.36 were significant. These findings suggest that the relationship between early morphine therapy and 30-day death varied significantly between the groups (Fig. 3).

## Discussion

This study reveals that for septic patients with CHF, administering morphine within the first 24 h is linked to a reduction in mortality at both 30-days and 90-days. It also results in a shorter duration of stay in the ICU and the hospital. Moreover, even after adjusting for confounding factors, this finding remains consistent in PSM analysis. The outcomes are also stable across various subgroups, including those with early propofol use, without fentanyl or midazolam use, regardless of chronic pulmonary disease presence, septic shock, mechanical ventilation, subsequent morphine treatment, and any Sequential Organ Failure Assessment (SOFA) score. Nevertheless, the correlation is not significant among subgroups with early fentanyl or midazolam use, or without propofol. These results suggest that early morphine therapy with a dose of <15 mg has a viable beneficial effect in septic patients with CHF, which has not been previously reported.

Many patients with CHF use morphine for a long time.^13^ It is even said that morphine is the dominant position in the treatment of chronic heart failure.^14^ Some studies have proved that the use of morphine in CHF can significantly relieve symptoms.^15^^,^^16^ However, most guidelines for CHF do not recommend the use of morphine because it has many side effects and can be replaced by other drugs with fewer side effects.^17^ Morphine is not recommended unless the patients have dyspnoea, anxiety, or the vasoconstriction accompanying hypertensive crises.^17^ Morphine is associated with increased mortality compared with midazolam in acute cardiogenic pulmonary edema.^18^ Nevertheless, randomized controlled studies have also shown that patients experiencing dyspnea may benefit from consistent, low-dose oral sustained-release morphine administered orally for four weeks; these trials have not shown deleterious effects on respiratory function and have not resulted in any hospital fatalities attributable to morphine use.^19^ Therefore, the use of morphine in patients with heart failure remains contradictory.

In clinical work, the authors also frequently use morphine in septic patients for various reasons.^20^^,^^21^ However, there are no adequate studies on the safety of morphine in patients with sepsis. Animal studies have shown that morphine in mice shifts the gut microbiota, and the mice became hypersusceptible to sublethal endotoxin challenge.^22^ The use of opioids is closely related to the occurrence of sepsis.^23^ Chronic morphine administration has also been shown to reduce endotoxin tolerance in humans, leading to prolonged inflammation, septicemia, and even septic shock.^10^ Whether it is for these reasons that morphine increases mortality in acute cardiogenic pulmonary edema patients in randomized controlled clinical trials requires further study.^18^ However, in this study, among patients in the early midazolam group, early morphine administration did not affect 30-day mortality. In patients who did not receive midazolam, early use of morphine was associated with reduced 30-day mortality.

Many patients with sepsis have underlying medical conditions, and CHF is one of the most common chronic diseases.^2^ Patients with sepsis and heart failure have multi-organ dysfunction, including myocardial depression and worsening systolic function.^24^^,^^25^ Several studies have demonstrated that sepsis patients with a history of CHF have a much higher mortality rate than those without CHF.^3^^,^^26^^,^^27^ Currently, there is no empirical guidance on the use of morphine in patients with sepsis and CHF. However, the present study shows that early use of morphine was associated with reduced 30-day mortality. Although early morphine use was a protective factor for death, later morphine use was a risk factor for death. In septic patients with CHF, is morphine appropriate only for short-term use, but not for long-term use? Further basic and clinical studies are needed to confirm the role of morphine in septic patients with a history of CHF.

Although, the use of morphine during mechanical ventilation in septic patients with a history of CHF has not been reported in the literature. However, there have been many studies on the use of morphine for the treatment of patients with mechanical ventilation due to various reasons, and most studies do not support the use of morphine.^28^^,^^29^ In this study, early use of morphine was associated with a significant reduction in 30-day mortality among patients who were not receiving mechanical ventilation, but it was associated with a more substantial decrease in 30-day mortality among those who received mechanical ventilation.

Although morphine has been used for many years, there is still no authoritative recommendation on the dose and duration of morphine use. Several randomized controlled studies have shown that after one week of therapy, individuals with severe chronic dyspnea and COPD who were taking daily low-dose (no >16 mg) extended-release morphine did not substantially lower the severity of worst breathlessness.^30^^,^^31^ Conversely, low-dose (20 mg or 30 mg daily) morphine helped COPD patients without changing PaCO_2_ or having serious side effects, according to a randomized clinical study.^19^ A case sharing concluded that morphine at a low dose of 2.5 mg/day improved not only the patient's dyspnea but also heart failure congestion, with an improvement in plasma BNP levels.^32^ The most appropriate dose of morphine remains unclear because of the wide range of morphine doses in these studies. In this study, early use of morphine at a dose of <15 mg improved mortality in patients with sepsis and underlying CHF.

The present study is the first of its kind to investigate the link between early morphine therapy and 30-day mortality in a large cohort of septic patients with a history of CHF. In addition, the authors delved into the risk factors associated with 30-day mortality and analyzed the impact of different morphine dosages on the first day's mortality rate. However, the present study is not without limitations. Firstly, due to its retrospective nature, the authors could not entirely eliminate the influence of confounding variables on mortality. A number of test results and risk factors, such as Brain Natriuretic Peptide (BNP), right heart function index, and Central Venous Pressure (CVP), were either not available or excluded due to a significant amount of missing data. This may have introduced bias and affected the comparability between the two groups. To mitigate the impact of confounding factors, the authors employed multivariate Cox regression and Propensity Score Matching (PSM) to balance as many comorbidities and other characteristics as possible between the groups. Secondly, this analysis did not account for other potential benefits of morphine, such as anxiety reduction and relief of respiratory symptoms. Thirdly, given that the present findings are based on data from a single-center database, caution should be exercised when interpreting these results and considering their applicability in real-world settings. Lastly, the authors did not assess the adverse effects of morphine, nor did we explore the relationship between early morphine use and the incidence of tracheal intubation and mechanical ventilation.

## Conclusion

In conclusion, the cohort study findings indicate a potential association between early administration of morphine prescriptions and a decrease in risk-adjusted mortality rates at 30 days among septic patients with CHF. This association appears to be particularly significant in-patient subgroups characterized by early use of propofol, absence of fentanyl and midazolam treatments, irrespective of the presence of chronic pulmonary disease, septic shock, mechanical ventilation status, subsequent morphine agent use, any SOFA score. These results suggest that early morphine administration could play a beneficial role in the management of septic patients with CHF, warranting further investigation to understand the mechanisms and potential clinical implications of this association.

## Authors' contributions

Zou Zhiye: Conceptualization; methodology; data curation, formal analysis; writing-original draft preparation; literature retrieval.

Gui Shuiqing: Conceptualization; writing-review and editing; supervision.

## Ethics approval and consent to participate

This study was approved by the Research Ethics Committee of Shenzhen Second People's Hospital (2024-145-01PJ). Considering the retrospective study design and depersonalization of the data, the Ethics Committee agreed to waive the requirement for written informed consent.

## Funding

This work was supported by grants from the Sanming Project of Medicine in Shenzhen (SZSM202211016), Shenzhen Fund for Guangdong Provincial High-level Clinical Key Specialties (No. SZGSP006), 10.13039/501100010877Shenzhen Science and Innovation Commission - 2020 Key Project of Technology Research (Innovation and Entrepreneurship) (No. JSGG20191118161401741).

## Data availability

Publicly available datasets were analyzed in this study. This data can be found at https://mimic.physionet.org.

## Declaration of competing interest

The authors declare that they have no financial or personal relationships with any individuals or organizations that could inappropriately influence the content of this manuscript. There are no competing interests to disclose, including employment, consultancies, stock ownership, honoraria, paid expert testimony, patent applications/registrations, or funding from grants.

## Appendix Supplementary materials

Image, application 1
