# Supplementary material for: The impact of early morphine administration on septic patients with pre-existing chronic heart failure
Source: Clinics (Sao Paulo). 2025 Apr 24;80:100655. doi: 10.1016/j.clinsp.2025.100655 (PMC12060473; doi:10.1016/j.clinsp.2025.100655)

**CLINICS-D-24-00660_Supplementary Material**

**Table S1** Missing number (%) for risk variables and outcome variables.

| **Risk variables** | **Missing number^a^ (%)** |
| --- | --- |
| Baseline characteristics | 0 (0) |
| Age (year) | 0 (0) |
| Male | 0 (0) |
| White | 0 (0) |
| Insurance, Medicare | 0 (0) |
| Weight (kg) | 99 (1.3) |
| Admission (emergency) | 0 (0) |
| History of disease |  |
| Hypertension | 0 (0) |
| Myocardial infarction | 0 (0) |
| Diabetes | 0 (0) |
| Renal disease | 0 (0) |
| Chronic pulmonary disease | 0 (0) |
| Charlson comorbidity index | 0 (0) |
| Infection sites |  |
| Respiratory infection | 0 (0) |
| Urinary tract infection | 0 (0) |
| Bloodstream infection | 0 (0) |
| Abdominal infection | 0 (0) |
| Vital signs at 1^st^ day |  |
| Mean MAP (mmHg) | 6 (0.1) |
| Maximum heart rate (bpm) | 5 (0.1) |
| Maximum respiratory rate (bpm) | 6 (0.1) |
| Laboratory outcomes at 1^st^ day |  |
| Maximum white blood cell (10^9/L) | 14 (0.2) |
| Maximum platelets(10^9/L) | 15 (0.2) |
| Septic shock | 0 (0) |
| Scoring system at 1^st^ day |  |
| Maximum SOFA score | 0 (0) |
| Maximum SAPII score | 0 (0) |
| Minimum GCS score | 0 (0) |
| In-hospital management at 1^st^ day |  |
| Mechanical ventilation | 0 (0) |
| Renal replacement therapy | 0 (0) |
| In-hospital medication at 1^st^ day |  |
| Furosemidum | 0 (0) |
| Dexmedetomidine | 0 (0) |
| Fentanyl | 0 (0) |
| Midazolam | 0 (0) |
| Propofol | 0 (0) |
| Later morphine agent | 0 (0) |
| Minimum LVEF | 2513 (33.8) |
| **Outcome variables** |  |
| 30-day mortality | 0 (0) |
| In-hospital mortality | 0 (0) |
| 90-day mortality | 0 (0) |
| Length of ICU stay (days) | 0 (0) |
| Length of hospital stay (days) | 0 (0) |

MAP, Mean Blood Pressure; bpm, beat per minute or breaths per minute; SOFA, Sequential Organ Failure Assessment; SAPII, Simplified Acute Physiology score II; LVEF, Left Ventricular Ejection Fraction; ICU, Intensive Care Unit.

^a^ The proportion of missing values is less than 2%. Multiple imputation was used to replace these missing values. Multiple imputation was not performed because more than 20% of the LVEF was missing.

**Table S2** Risk factors of 30-day all-cause mortality in septic patients with a history of CHF.

|  | **Univariable** | | | **Multivariable^a^** | | |
| --- | --- | --- | --- | --- | --- | --- |
| **Variables** | **HR** | **95% CI** | **p-value** | **HR** | **95% CI** | **p-value** |
| Baseline characteristics |  |  |  |  |  |  |
| Age | 1.032 | 1.027‒1.037 | <0.001 | 1.026 | 1.019‒1.034 | <0.001 |
| Male | 1.000 | 0.904‒1.107 | 0.997 |  |  |  |
| White | 0.867 | 0.781‒0.963 | 0.008 | 0.945 | 0.819‒1.090 | 0.437 |
| Insurance, Medicare | 1.272 | 1.144‒1.413 | <0.001 | 0.988 | 0.855‒1.142 | 0.871 |
| Weight | 0.990 | 0.988‒0.993 | <0.001 | 0.994 | 0.991‒0.997 | <0.001 |
| Admission (emergency) | 1.035 | 0.936‒1.144 | 0.506 |  |  |  |
| History of disease |  |  |  |  |  |  |
| Hypertension | 1.107 | 0.972‒1.261 | 0.125 |  |  |  |
| Myocardial infarction | 1.303 | 1.174‒1.445 | <0.001 | 1.073 | 0.927‒1.243 | 0.342 |
| Diabetes | 0.941 | 0.850‒1.041 | 0.236 |  |  |  |
| Renal disease | 1.374 | 1.243‒1.520 | <0.001 | 0.920 | 0.771‒1.097 | 0.352 |
| Chronic pulmonary disease | 0.977 | 0.882‒1.082 | 0.655 |  |  |  |
| Charlson comorbidity index | 1.171 | 1.145‒1.197 | <0.001 | 1.100 | 1.054‒1.149 | <0.001 |
| Infection sites |  |  |  |  |  |  |
| Respiratory infection | 1.744 | 1.576‒1.930 | <0.001 | 1.242 | 1.079‒1.428 | 0.002 |
| Urinary tract infection | 1.045 | 0.924‒1.181 | 0.487 |  |  |  |
| Bloodstream infection | 1.422 | 1.212‒1.668 | <0.001 | 1.008 | 0.816‒1.245 | 0.941 |
| Abdominal infection | 1.344 | 1.102‒1.638 | 0.003 | 1.036 | 0.799‒1.150 | 0.777 |
| Vital signs at 1^st^ day |  |  |  |  |  |  |
| Mean MAP (mmHg) | 0.987 | 0.982‒0.993 | <0.001 | 0.998 | 0.991‒1.006 | 0.642 |
| Maximum heart rate (bpm) | 1.007 | 1.005‒1.009 | <0.001 | 1.003 | 1.000‒1.007 | 0.029 |
| Maximum respiratory rate (bpm) | 1.023 | 1.015‒1.030 | <0.001 | 1.021 | 1.011‒1.031 | <0.001 |
| Laboratory outcomes at 1^st^ day |  |  |  |  |  |  |
| Maximum white blood cell (10^9/L) | 1.019 | 1.013‒1.024 | <0.001 | 1.010 | 1.002‒1.019 | 0.015 |
| Maximum platelets(10^9/L) | 1.000 | 1.000‒1.000 | 0.230 |  |  |  |
| Septic shock | 1.731 | 1.565‒1.915 | <0.001 | 1.028 | 0.856‒1.235 | 0.767 |
| Scoring system at 1^st^ day |  |  |  |  |  |  |
| Maximum SOFA score | 1.150 | 1.136‒1.165 | <0.001 | 1.124 | 1.088‒1.162 | <0.001 |
| Maximum SAPII score | 1.043 | 1.039‒1.046 | <0.001 | 1.008 | 1.001‒1.015 | 0.027 |
| Minimum GCS score | 0.881 | 0.871‒0.890 | <0.001 | 0.953 | 0.934‒0.973 | <0.001 |
| In-hospital management at 1^st^ day |  |  |  |  |  |  |
| Mechanical ventilation | 1.208 | 1.093‒1.336 | <0.001 | 0.639 | 0.507‒0.806 | <0.001 |
| Renal replacement therapy | 1.673 | 1.454‒1.924 | <0.001 | 1.234 | 1.009‒1.510 | 0.041 |
| In-hospital medication at 1^st^ day |  |  |  |  |  |  |
| Furosemidum | 0.910 | 0.821‒1.009 | 0.075 | 1.137 | 0.991‒1.306 | 0.067 |
| Dexmedetomidine | 0.702 | 0.543‒0.907 | 0.007 | 0.788 | 0.555‒1.118 | 0.182 |
| Fentanyl | 1.352 | 1.221‒1.496 | <0.001 | 1.024 | 0.817‒1.285 | 0.835 |
| Midazolam | 1.488 | 1.327‒1.668 | <0.001 | 1.013 | 0.813‒1.262 | 0.908 |
| Propofol | 0.764 | 0.686‒0.850 | <0.001 | 0.709 | 0.587‒0.856 | <0.001 |
| Later morphine agent | 3.344 | 3.007‒3.718 | <0.001 | 2.875 | 2.473‒3.344 | <0.001 |
| Minimum LVEF | 0.986 | 0.980‒0.991 | <0.001 | 0.989 | 0.984‒0.995 | <0.001 |
| Early morphine use | 0.591 | 0.491‒0.711 | <0.001 | 0.544 | 0.418‒0.707 | <0.001 |

HR, Hazard Ratio; CI, Confidence Interval; MAP, Mean Blood Pressure; bpm, beat per minute or breaths per minute; SOFA, Sequential Organ Failure Assessment; SAPII, Simplified Acute Physiology score-II; GCS, Glasgow Coma Scale; LVEF, Left Ventricular Ejection Fraction.

^a^ First, a univariate analysis was performed. Then variables were selected for inclusion in multivariate analysis based on p-value less than 0.1.

**Figure S1** Standardized Mean Difference (SMD) of variables before and after propensity score matching.


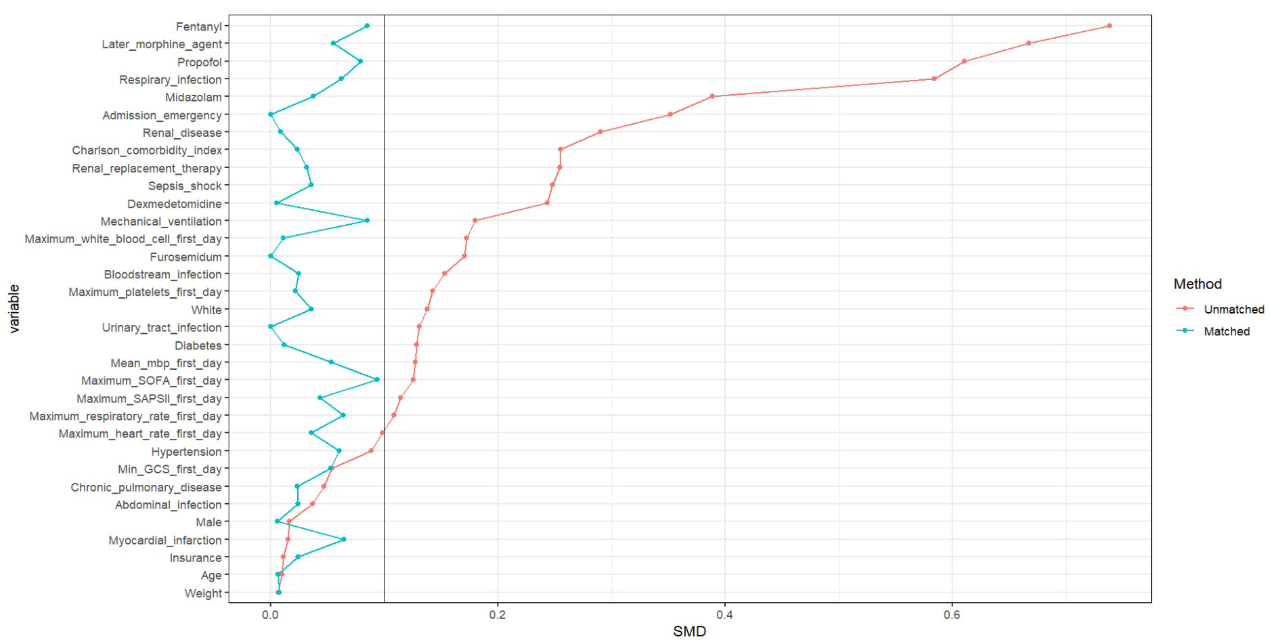

Supplement: Supplementary file 1 [file mmc1.docx]
